# Immediate rotationplasty for a severely crushed floating knee in a blast injury: A case report

**DOI:** 10.1016/j.tcr.2021.100600

**Published:** 2021-12-28

**Authors:** Chun-Kuan Lu, Ying-Chun Liu, Chih-Ting Chen, Yin-Chih Fu, Wen-Chih Liu

**Affiliations:** a Department of Orthopedic Surgery, Park One International Hospital, Kaohsiung, Taiwan; b School of Post-Baccalaureate Medicine, College of Medicine, Kaohsiung Medical University, Kaohsiung, Taiwan; c Department of Orthopedic Surgery, Kaohsiung Medical University Hospital, Kaohsiung, Taiwan; d Department of Orthopedic Surgery, Kaohsiung Municipal Ta-Tung Hospital, Kaohsiung, Taiwan; e Ph.D Program in Biomedical Engineering, College of Medicine, Kaohsiung Medical University, Kaohsiung, Taiwan

**Keywords:** Rotationplasty, Limb salvage, Floating knee, Knee crushing injury, Trauma

## Abstract

Rotationplasty is a durable biological reconstruction strategy that is most often performed in children with osteosarcoma of the distal femur. This limb-sparing procedure essentially employs a 180° “rotation” of the distal limb followed by fixation to the proximal limb, resulting in superior functionality and flexibility as compared to those of alternative surgeries. However, despite the many advantages of rotationplasty, literature regarding its indications, techniques, and outcomes in adult patients is scarce. A 37-year-old man presented with a severely floating knee in a blast injury. In addition to femoral shaft fracture, the proximal tibia was comminuted severely from the articular surface to the diaphysis, and the soft tissue was equally crushed. Because his ankle was relatively intact, immediate rotationplasty was performed for joint reconstruction combined with anastomosis of the neurovascular bundles. He underwent another bone grafting surgery 8 months after the initial surgery to improve bone union and subsequently began full weight-bearing with a prosthesis 3 months later. After more than 4 years of follow-up, he could walk without assistance, was satisfied with his overall recovery, and had a decent range of motion. However, due to the injured tibial nerve from the initial accident, he continued to experience numbness of the left foot, which prevented him from wearing the prosthesis for more than 3 h at a time. Based on our experience and literature review, opting for rotationplasty after a trauma will provide optimal outcome for the patient only when the following conditions are met: (1) healthy and active preoperative status, (2) integrity of the nerves, (3) competence of the prosthetic team, and (4) access to an emergency microsurgical reconstruction trauma center facility.

## Introduction

Rotationplasty is a limb-sparing procedure involving 180° rotation of the ankle, allowing it to function as a knee joint. It was first described in 1930 by Borggreve, and Van Nes popularized it in 1950 for treating congenital defects of the femur. In 1981, Salzer used rotationplasty to treat osteosarcoma of the distal femur, and to date, it is the standard indication for this condition [1].

When first introduced, the benefit of rotationplasty was evident in skeletally immature patients, mainly because it helped in avoiding phantom pain and further operations to correct limb length discrepancy due to growth [2]. Furthermore, compared with above-the-knee (AK) amputation, rotationplasty is reported to yield outstanding functional outcomes by allowing the patients to maintain limb length, enabling a more active and efficient gait with less energy consumption during ambulation and less postoperative pain. These benefits result in a better quality of life [3].

Despite its many advantages, rotationplasty is rarely indicated in adult patients with trauma. To our knowledge, only a few case reports have described posttraumatic limb salvage with rotationplasty [4], [5], [6]. Herein, we present a case of severe knee injury treated by immediate rotationplasty.

## Case report

**Fig. 1:**
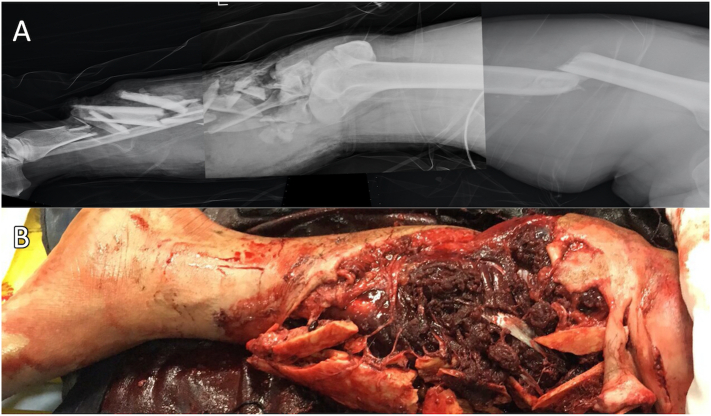
(A) The radiograph shows the femur shaft fracture and ipsilateral tibial plateau articular comminuted fracture extended to the diaphysis. (B) A large soft tissue defect around the knee is evident.

A 37-year-old healthy male migrant worker experienced a gaseous explosion while cutting gas barrels. He was sent to the emergency department with a nearly amputated leg above his left knee and in hypovolemic shock. After resuscitation, radiographs demonstrated type IIIC proximal tibial intra-articular and metaphyseal comminuted open fractures and left femoral shaft transverse fractures (Fig. 1). Following the primary and secondary surveys, the microsurgical reconstruction team was activated for emergency surgery.

**Fig. 2:**
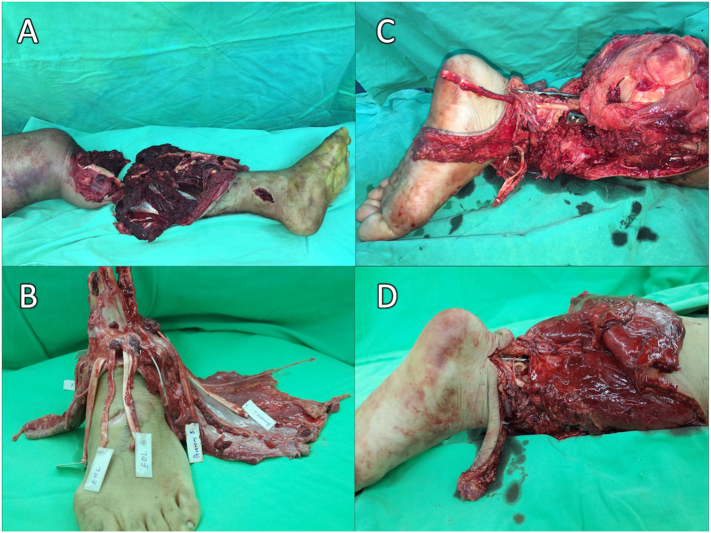
(A) The leg is completely amputated after debridement. (B) The main structure was tagged and prepared for repair. (C) The tibial stump was inserted into the femoral canal and fixed with a conventional plate. We established the circulation by anastomosing the plate posterior artery and its concomitant vein to the popliteal artery and its concomitant vein.

After debridement, the limb was amputated entirely (Fig. 2A). Considering that the left ankle joint was uninjured and that he was young and active, knee rotationplasty could provide him with a better functional outcome. The microsurgical reconstruction team was divided into two sub-teams to identify and tag the arteries, veins, nerves, and tendons of the stump and amputee (Fig. 2B). An osteotomy was performed at the distal femur, with the distal tibia inserted into the femoral canal. Under meticulous measurement, the level of the left ankle joint matched that of the right knee joint. Subsequently, the posterior tibial artery was anastomosed to the popliteal artery, concomitant vein of posterior tibial artery was anastomosed was to the popliteal vein and the great saphenous vein anastmosis (Fig. 2C). After restoring the circulation, the proximal end of the tibial nerve was coated to the distal stump of the tibial nerve and the sural nerve at the ankle stump. Lastly, the ankle dorsiflexor tendons were repaired to the knee flexor tendons, and the ankle plantar flexor tendons were repaired to the knee extensor tendons using interweaving techniques (Fig. 2D).

**Fig. 3:**
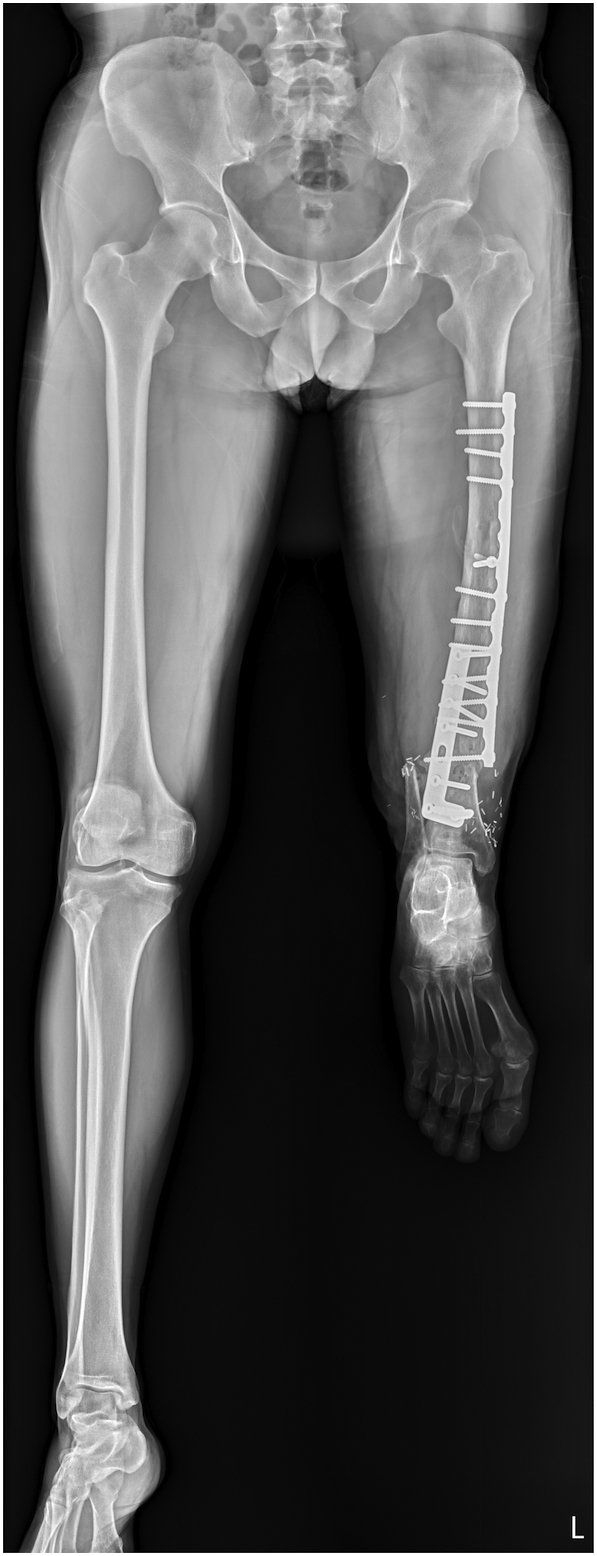
Whole leg axis radiographs. The bone union site has completely healed, and the axis of the leg is straight.

Eight months after the initial injury, nonunion was detected at the femur-tibia site. The 3.5-mm straight implant plate was removed and exchanged for a 4.5-mm L-shape plate with morcellated allografts inserted in the bony canal to increase mechanical strength and union rate. He began full weight-bearing with a prosthesis 3 months after the second surgery, and at that time, plain radiographs showed union at the osteosynthesis site. The prosthetist embraced a custom-made additive-manufactured permanent orthosis 7 months after the second operation. The radiograph showed complete union at the osteosynthesis site and a straight axis of the entire leg (Fig. 3). He received standard postoperative physical therapy sessions three times per week for one year. Physical therapy included muscle stimulation, strength training, isokinetic and endurance training, as well as low power laser, infrared, and transcutaneous electrical nerve stimulation therapy. Two years after the initial injury, the patient returned to his home country. He continued the home-based exercise plan on his own.

**Fig. 4:**
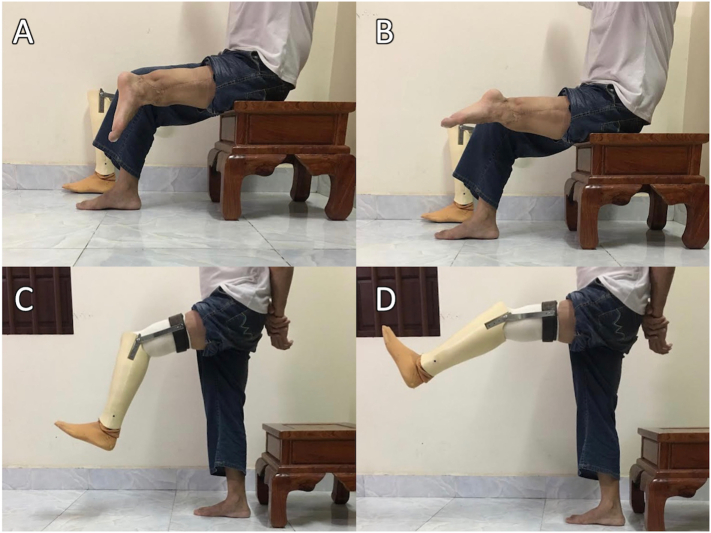
(A) Range of motion of the ankle joint: maximum plantar flexion (70°). (B) Range of motion of the ankle joint: maximum dorsiflexion (0°). (C) Range of motion of the prosthetic knee joint: maximum flexion (60°). (D) Range of motion of the prosthetic knee joint: 15° of extensor lag.

At the most recent follow-up in a virtual clinic over 4 years after rotationplasty, he could ambulate independently with a prosthesis. The active range of motion of the left ankle joint was 0°–70°, and that of the prosthetic knee was 15°–60° (Fig. 4). His visual analog pain score was 2 on a scale of 10, and his range of motion was relatively satisfactory. However, the injured tibial nerve caused left-foot numbness that prevented walking for longer than 3 h at a time.

## Discussion

Rotationplasty is most commonly indicated for malignant tumors of the knee in children. Despite some cosmetic concerns, it has been proven far superior to other alternatives because its high functionality and low complication rates allow patients to live a relatively “normal” life [1]. Several studies have documented good functional outcomes in patients after rotationplasty in long-term follow-ups [7], [8].

Nonetheless, rotationplasty is rarely considered for adults with severe trauma. Only three adult patients have undergone knee rotationplasty after trauma, and two of the three patients initially experienced severe infection [4], [5]. In 1997, Krettek described a young male patient with a type IIIC, AO 33-C3.3 open fracture who sustained a severe infection after the initial revascularization surgery and received a Winkelmann AI rotationplasty [4]. This surgery provided the 31-year-old carpenter a chance to repurpose his uninjured ankle joint to function as an alternative knee joint. At the patient's 18-year follow-up, his gait analysis score and SF-36 questionnaire were equivalent to those of normal individuals, and some results of the questionnaire were even better [6].

Klos et al. reported an 80-year-old elder with serious de-gloving injuries on his left hemipelvis [5] for which he underwent a Winkelmann AI rotationplasty. However, the postoperative outcomes were unsatisfactory. The range of motion of the ankle joint remained limited at the 2-year follow-up.

The most recent case was described by Tye et al. [9] A 26-year-old man presented with a Type IIIA open femur fracture complicated by an intractable necrotizing soft-tissue infection and a 12-cm segment of bone loss. He received a Winkelmann AI rotationplasty with preservation of the common peroneal, tibial, and sciatic nerves. He was fitted for a prosthesis at 4 months postoperatively and walked independently at 13 months. No recurrent infection was found after the 3-year follow-up.

The major difference between our case and the three cases described above is that our patient received a one-stage Winkelmann AII rotationplasty promptly after the initial injury. Although a bone grafting surgery due to nonunion detection was necessary 8 months after rotationplasty, in contrast to choosing revascularization and further complicated knee reconstruction surgery, his hospitalization and recovery time was relatively short. In the following section, we present lessons learned from this case after 4 years of follow-up.

First, mental support is crucial in guiding patients to achieve psychosocial well-being. Due to his severe injury and shock, our patient initially underwent surgical resuscitation, and many medical decisions were made by his advocates, not by himself. At the time, the only other option was AK amputation because the explosion made limb salvage surgery with endoprosthetic replacement impossible. Therefore, a shared decision-making process was undertaken. Given his active pre-surgery status and intact ankle joint, his medical advocates and the medical team agreed to perform knee rotationplasty. However, after he recovered from anesthesia, he did not understand the rationale for receiving a rotationplasty and insisted on having the reversed ankle joint removed. It required great efforts and much time for the medical team to counsel him, helping him to eventually fully embrace his new “knee.” Ideally, it is preferred to have an exhaustive pre-surgery consultation session with the patient to ensure consent beforehand, although this was not possible in our case.

Second, it is critical to have a competent prosthetic team. Owing to the relatively conservative sentiment in Taiwan, rotationplasty is rarely performed. Even malignant bone tumors were treated with rotationplasty mostly in the early 1990s [10]. Therefore, local prosthetists have few comparable experiences. In our case, the soft socket did not fit the patient's foot well; therefore, the initially available prosthesis employed a considerably thick belt tied to the thigh, with a heavy metal stick connecting the thigh to the distal prosthesis. This prosthetic design was meant for AK amputation, making it very bulky for the patient. Later, we located a prosthetist who was willing to pioneer using an additive-manufactured technique to custom-make a new set of prostheses, enabling better gait outcomes and comfort for him.

Third, the institute where the author is based is a territory trauma center, with a 24-hour on-call microsurgical reconstruction team, including orthopedic and plastic hand surgeons. The team was divided into two sub-teams to prepare the stump and amputee. The repair proceeded efficiently and in an orderly manner by tagging the essential neurovascular structure and tendons, shortening the ischemic time, and thus rendering a successful surgery. As the patient presented with a comminuted fracture with truncated vessels, all major vessels were truncated and re-anastomosed by the microsurgical team. This procedure is aligned with a literature review presented by Fujiki et al. [11] showing that en bloc resection reduces the possibility of vascular compromise and the risk of kinking observed in the conventional Van Nes rotationplasty approach, where the neurovascular structures are instead coiled. On the other hand, the main disadvantage of transection and reanastomosis of the vessels is the risk of anastomotic failure, which is approximately 12% [12]. Although the reasons for vascular compromise might vary, having a competent and experienced microsurgical team to ensure successful anastomoses without leakage is important.

Finally, nerve integrity is imperative in rotationplasty. Traditionally, when performing rotationplasty, nerves were preserved wholly by coiling and were hidden in the medial side of the osteotomy, thereby avoiding neurologic symptoms such as phantom pain or any neuropathic sensations [13]. However, our patient's tibial nerve was injured and truncated by the initial trauma. Therefore, during follow-up, he continued to complain about the numbness of his entire foot and neuropathic sensations that negatively affected his willingness to walk long distances. We hypothesized that the lack of physiotherapy and medical treatment after he returned to his hometown might be a reason for the persistent ailments observed at the 4-year follow-up.

Given that modern prosthesis techniques are quite advanced, if our patient had chosen AK amputation followed by bionic reconstruction combined with the beneficial effects of targeted muscle innervation and regenerative peripheral nerve interface [14], a similar functional effect to rotationplasty might have been achieved. However, not every patient has the same access to cutting-edge technology or the ability to afford it. In addition, one study demonstrated a difference in cortical and subcortical reorganization between above-knee amputees and rotationplasty patients [15]. Without the afferent input from the amputation, reorganization of the contralateral sensorimotor regions of the brain occurs. As a result, lower limb amputees have decreased functional connectivity between the contralateral sensorimotor cortex and subcortical motor regions. In properly selected patients, rotationplasty may still provide superior functional outcomes than AK amputation.

In conclusion, when encountering patients with severe knee trauma, one must evaluate the physical status, competence of the prosthetics team, facility of the emergency microsurgical reconstruction trauma center, and nerve integrity. Only when these conditions are aligned, rotationplasty performed after severe trauma will provide favorable outcomes for the patient.

## Ethics approval and consent to participate

The patients agreed to the publication of their surgical images. This research project was analyzed and approved by the Institutional Review Board of Kaohsiung Medical University Hospital (KMUHIRB-E(I)-20210104).

## Authors' contribution

Each author is expected to have made substantial contributions to the conception. CKL and WCL designed of the work; YCL and CTC made the data acquisition and analysis; CKL and WCL made the data interpretation; CKL and CYL have drafted the work; YCF and WCL substantively revised it. All the authors approved the final version to be submitted.

## Declaration of competing interest

The authors declared no potential conflicts of interest with respect to the research, authorship, and/or publication of this article.
